# Label and Label-Free Detection Techniques for Protein Microarrays

**DOI:** 10.3390/microarrays4020228

**Published:** 2015-04-24

**Authors:** Amir Syahir, Kenji Usui, Kin-ya Tomizaki, Kotaro Kajikawa, Hisakazu Mihara

**Affiliations:** 1 Department of Biochemistry, Faculty of Biotechnology and Biomolecular Science, Universiti Putra Malaysia, Serdang 43400, Malaysia; 2 Faculty of Frontiers of Innovative Research in Science and Technology (FIRST), Konan University, Kobe, Hyogo 650-0047, Japan; 3 Department of Materials Chemistry, Ryukoku University, Seta, Otsu 520-2194, Japan; E-Mail: tomizaki@rins.ryukoku.ac.jp; 4 Department of Electronics and Applied Physics, Interdisciplinary Graduate School of Science and Engineering, Tokyo Institute of Technology, Yokohama, Kanagawa 226-8502, Japan; E-Mail: kajikawa@ep.titech.ac.jp; 5 Department of Bioengineering, Graduate School of Bioscience and Biotechnology, Tokyo Institute of Technology, Yokohama, Kanagawa 226-8501, Japan; E-Mail: hmihara@bio.titech.ac.jp

**Keywords:** protein microarray, protein chip, detection method, label technique, label-free technique

## Abstract

Protein microarray technology has gone through numerous innovative developments in recent decades. In this review, we focus on the development of protein detection methods embedded in the technology. Early microarrays utilized useful chromophores and versatile biochemical techniques dominated by high-throughput illumination. Recently, the realization of label-free techniques has been greatly advanced by the combination of knowledge in material sciences, computational design and nanofabrication. These rapidly advancing techniques aim to provide data without the intervention of label molecules. Here, we present a brief overview of this remarkable innovation from the perspectives of label and label-free techniques in transducing nano-biological events.

## 1. Introduction

Microarrays are now recognized as powerful tools for predictive and early disease diagnosis, as well as research in many biological fields. In each microarray, also known as ‘biochips’, thousands of reaction spots (capturing agent spots) are arrayed onto a few square centimeters. It is a remarkably versatile and useful platform, particularly when dealing with very limited sample volumes in genomics [1], proteomics [2,3,4], transcriptomics [5,6], glycomics [7,8,9], metabolomics [10], and cellomics [11] research. Partially automated dispensing systems, condition controlling systems and automated signal readouts enable sophisticated experiments to be carried out by non-specialized technicians.

Protein microarrays are mainly constructed to achieve two primary objectives [12,13]: (i) to identify and quantify protein abundance, which has been applied in a range of cancer studies, specifically in screening and searching for biomarkers and protein drugs, as well as the diagnosis of particular cancers by distinctive fingerprints; and (ii) to study protein (or biomolecular) functions, which involves the intermolecular interactions that reveal new insight into molecules of interest or/and important characteristics such as binding parameters, enzymatic functions and novel biomolecular interactions. Consequently, numerous protein microarrays have been developed. In addition to DNA/RNA microarrays [14] and whole protein microarrays, such as antibody/antigen microarrays [15] and whole-proteome scale microarrays [16], small molecule microarrays [17] and peptide microarrays including α-helical peptides [18,19], β-turn peptides [20], sugar modified peptides [21], and peptides from protein sequences [22], are also the promising tools for protein analysis.

Protein microarrays are supported by three key technologies: (i) production of functional capture agents; (ii) surface chemistry; and (iii) development of high-throughput detection methods. These technologies must be tied with one another in order to provide valuable protein detection. To date, dozens of exciting reviews on protein microarrays have been reported (for example [12,13,23,24,25,26]); these reviews have already described the concepts of such microarray technologies and have focused on the production of microarrays and the development of capture agents. Meanwhile, we have designed peptide libraries composed of various secondary structures to use for protein microarray technologies, and in parallel, novel techniques for detection have been also developed. In this mini-review, we focus on the “new and old” technologies for detection in protein microarrays. We also present a brief overview of these remarkable innovations from the perspective of label and label-free techniques in transducing nano-biological events.

## 2. “New and Old” Detection Technologies

A good detection method that can be applied to microarray usage generally offers: (i) high signal-to-noise ratio; (ii) high spatial resolution; and (iii) good reproducibility. While offering broad practicality, methods need to have low instrumentation costs, rapid determination and robustness, while producing good qualitative results. Although there is not yet a general sensing method that can be ubiquitously applied to detect the cluster of biomolecules in various samples, there are several candidates that can be considered.

In this review we focus on five candidate label detection methods, namely, (1) fluorescent labeling, (2) isotopic labeling, (3) chemiluminescent labeling, (4) electrochemically active probe labeling, and (5) nanoparticle labeling, and five candidate label-free detection methods, namely, (6) mass spectrometry (MS), (7) microcantilevers, (8) quartz-crystal microbalance (QCM), (9) surface plasmon resonance (SPR) and localized surface plasmon resonance (LSPR), and (10) anomalous reflections of the gold surface (AR). The features of the different methods with respect to their availability for protein microarrays are summarized and discussed below. Table 1 summarizes the main advantages and disadvantages of label and label-free approaches.

A label is defined as any foreign molecule that is chemically or temporarily attached to the molecule of interest to detect molecular presence or activity, which can potentially alter its intrinsic properties. It requires a labeling process as a preparation step that is usually low yield, combining synthesis and purification. Fluorescent, chemiluminescent, and nanoparticle labeling usually involves covalent bonding through coupling chemistries, while some of electrochemically active probe labeling requires only temporary attachment of intermolecular bonding. Isotopic labeling implicates “light” and “heavy” elements being incorporated into target molecules to result a detectable difference.

Meanwhile, label-free detection methods utilize molecular biophysical properties such as molecular weight (e.g., in microcantilever and MS), refractive index (e.g., in SPR, LSPR, and AR) and molecular charge to monitor molecular presence or activity. Furthermore, these methods can be used to track molecular events in a real-time manner. In a typical biosensing process, molecular interactions are transduced as mechanical, electrical, or optical signals, and are thus detectable without any label probes. The main advantage for label-free detection is that more direct information can be acquired, as the methods use only native proteins and ligands.

**Table 1:** Detection methods applicable to the development of protein microarray technology.

		Labeling/Preparation	Handling	Instrumentation Cost	Quantitative	High Throughput
**Labeling**	Fluorescent probe	Yes/Medium	Easy	Inexpensive	Yes/No	Yes
	Radioisotope	Yes/Difficult	Difficult	Medium	Yes	Yes/No
	Chemiluminescent probe	Yes/Medium	Easy	Inexpensive	Yes/No	Yes
	Electrochemical probe	Yes/Medium	Easy	Inexpensive	Yes	Yes/No
	Nanoparticles	Yes/Medium	Easy	Inexpensive	Yes	Yes
**Non-Labeling**	MS	No/Easy	Easy	Expensive	No	Yes/No
	Microcantilever	No/Difficult	Difficult	Expensive	Yes	No
	QCM	No/Medium	Easy	Inexpensive	Yes	No
	SPR	No/Medium	Easy	Expensive	Yes	Yes
	AR	No/Medium	Easy	Inexpensive	Yes	Yes

## 3. Label Detection

### 3.1. Fluorescent Labeling

Fluorescent label detection methods are the most common and convenient techniques to transmit information from molecular events. Fluorescence probes are stable, easily manipulated, and provide good sensitivity and resolution [27]. These are especially important when incorporated into microarray technology. Although perturbations in molecular interactions caused by label molecules can lead to false-positive signals [28], large amounts of data produced from high-throughput studies can be subjected into statistical data-mining processes that can increase analytical accuracy [29]. In an antibody sandwich assay system, a fluorescent dye molecule is used as a secondary antibody label. In this way, one can omit a direct non-native effect on the molecule of interest.

Fluorescence probes are currently available in many forms, from quantum dots and small organic molecules to fluorescent proteins with a range of brightness and specificity that can be selected based on need [30,31]. The emergence of advanced fluorescence techniques such as fluorescence resonance energy transfer (FRET), bimolecular fluorescence complementation (BiFC), and fluorescence correlation spectroscopy (FCS) has enabled the use of smaller samples (approx. 1 fL) with lower detection limits. We briefly cover FCS detection in this review. The method provides recording data for spatiotemporal correlations among fluctuating light signals coupled with the trapping of single molecules in an electric field. The diffusion times obtained from the fluctuating signals depend on the molecular sizes of the complex formed. Although the FCS assay system is expected to provide detailed information on molecular interactions on a proteome-wide scale, there are some disadvantages, such as strong dependence upon differences in molecular size before and after complexation and relatively low-throughput measurement [32,33,34].

**Figure 1:**
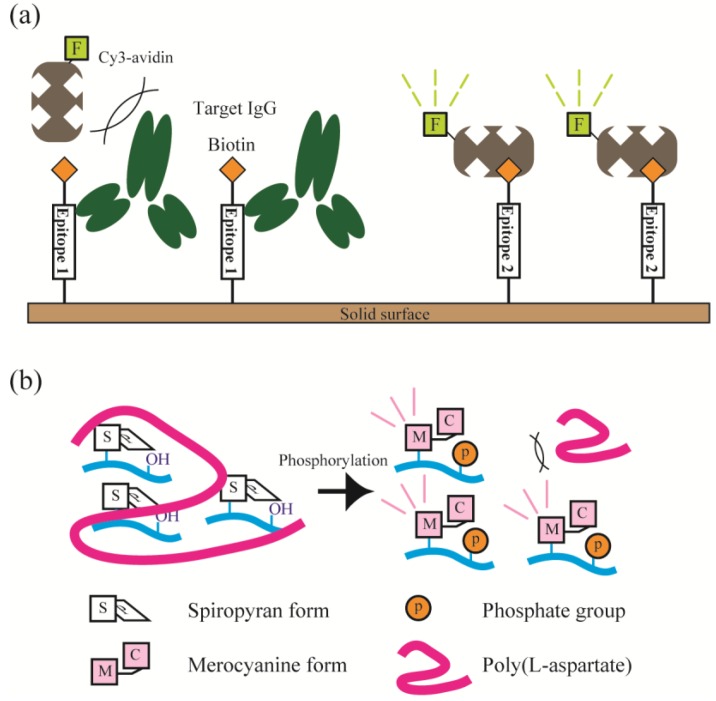
(**a**) Illustration of non-competitive on-chip immunoassays with target IgG (left) or without target IgG (right) for detection of non-labeled antibodies based on excluded volume effect of the target itself. (**b**) Illustration of homogenous protein kinase assay (chromism-based assay: CHROBA) platforms that allow both fluorometric and colorimetric signal readouts.

We also developed new fluorescent labeling detection methods utilizing the excluded volume effect of the target molecule or fluorometric/colorimetric changes in the detection moiety (Figure 1). In the excluded volume effect method (Figure 1a) [35], we proposed a noncompetitive and on-chip immunoassay format based on the excluded volume effect by a target antibody bound to its epitope on a microarray. This method is advantageous because only a biotin-conjugated peptide epitope (capture agent) and fluorophore-labeled-avidin (signal-generating agent) pair are required for signal readout. This concept would allow fine tuning of the excluded volume and binding rate to the capture agent for improvement of sensitivity and versatility. In the fluorometric/colorimetric change method (Figure 1b) [36,37,38], a chromism-based assay (CHROBA) technique using photochromic spiropyran-conjugated peptides was established for detection of kinase phosphorylation. This method allowed us to save isolation and/or substrate-immobilization steps to remove excess reagents including nonreactive isotope-labeled ATP or fluorescently-labeled anti-phosphoamino acid antibodies from the reaction solution.

### 3.2. Isotopic Labeling

One of the earliest methods for molecular detection involved the use of radioisotopes that enables accurate quantification of protein abundance [39]. Although radioactivity is also suitable for analyzing protein activity, particularly for enzymatic phosphorylation due to sensitivity and specificity, as well as the possibility of fluorescence detection [40], the use of isotope-labeled molecules raises some safety concerns [41,42]. Therefore, in the long run, these methods are not likely to be adopted for a broad range of biochemistry experiments.

Very recently, however, Ong *et al.* introduced stable isotope labeling using amino acids in cell cultures (SILAC) [43]. They reported the identification of methylation sites using SILAC, which does not require normal labeling or purification steps. This method is combined with mass spectroscopy to identify and quantify relative protein abundance between normal and isotope-labeled proteins [44].

### 3.3. Chemiluminescent Labeling

Chemiluminescence is another vibronic phenomenon that is useful to transduce molecular interactions into analyzable color information [45]. Obtaining its energy from exoergic reactions, different wavelengths are emitted during molecular relaxation to its ground state depending on the amount of energy acquired (e.g., 150 kJ/mol for red light, 300 kJ/mol for blue light). Szkola *et al.* recently demonstrated simultaneous detection of small and large molecules on microarray immunoassays [46]. They combined sandwich immunoassays and competitive immunoassays on a flow-through chemiluminescence microarray platform. The chemiluminescence signal was amplified using a poly-horseradish peroxidase complex (polyHRP), resulting in low detection limits; microgram or submicrogram levels for both small (<1 kDa) and large (>10 kDa) molecules. The utilization of chemiluminescent probes in microarray takes advantage of its high sensitivity and a dynamic range of up to six orders of magnitude [47]. However, the quantum yield of chemiluminescent probes is about 1% or lower, due to inefficiency in the chemical reaction or poor energy transfer [48].

### 3.4. Electrochemically Active Probe Labeling

Sensing of electrochemical signals originating from molecular surface charge has been reported for high-throughput studies [49,50]. This detection method is particularly attractive because of its sensitivity and robustness, and because it can be miniaturized. The Leiber group has pioneered the multiplex detection of prostate specific antigen (PSA), PSA-1-antichymotripsin, carcinoembryonic antigen, and mucin-1 (all are cancer biomarkers) at femtomolar concentrations using FET nanowire sensors [50]. Goda and Miyahara performed a miniaturized and multi-channeled detection of thrombin and lysozyme on extended-gate FET [51]. Esfandyarpour *et al.* measured changes of impedance using nanoneedle-sensing electrodes to detect the abundance of charged protein (strapavidin) at nM concentration [52].

Very recently, Das *et al.* reported a universal detection technique using the displacement method for electrochemically active probes (neutralizer displacement assay (NDA)) [53]. The NDA system utilizes a designed aptamer that loosely binds to the neutralizer, which later functions as a signal carrier. It induced clear 100 nA amperometric signal differences on adenosine triphosphate (ATP) detection. Although it is sufficiently sensitive to detect DNA at fM concentrations, manufacturing highly dense multichannel detection nano-electrodes that can independently function as individual reaction chambers remains a challenge. In the future, the multiplex detection format may be applied to lithographic techniques and suitable microchip design [54].

### 3.5. Nanoparticles: Macro-Labeling

The utilization of nanoparticles (NP) or metal nanoclusters in molecular detection is sometimes associated with label-free detection methods. However, <10 nm NP are considered to be probes that assist in molecular detection. The target protein is bound with the NP beforehand, and on molecular interaction, event signal monitoring is dependent on the NP signal [55]. Thus, it falls into another class of (macro-)label detection. It is reported that 1.4-nm gold nanoparticle probes that were covalently attached to antibodies improved immunodetection [56]. Direct molecular absorption to NPs may induce some molecular distortion that modifies its intrinsic function. To reduce this effect, a self-assembled monolayer (SAM) with suitable functional group is used as a bio-interfacial surface [57].

Integration of NP labeling (particularly gold NP) with SPR or Raman scattering measurements is useful for signal amplification because it absorbs more light energy from localizing resonance effects at a particular wavelength [58,59]. Cao *et al.* performed multiplexed detection in a microarray format with AuNP functionalized protein (12 spots/1 mm^2^) [60]. Recently, Li *et al.* studied intracellular kinase activity using AuNP probes in a peptide microarray [61]. The method, known as resonance light scattering (RLS) assay, consequently utilized Au NP probes as seeds for silver staining signal amplification.

## 4. Label-Free Detection

### 4.1. Mass Spectrometry (MS)

MS, including MS/MS, allows us to directly identify proteins of interest by means of molecular mass or mass patterns after tryptic digestion. However, conventional MS has some disadvantages and low throughput. Surface-enhanced laser desorption/ionization (SELDI) TOF-MS is an innovative approach that offers on-chip purification of the proteins of interest and subsequent ionization of the retained molecules to be detected [62]. SELDI-TOF-MS has been applied to screening for tumor biomarkers [63,64,65], plant phosphoproteome [66], and the detection of other proteins in a chip format. Yeo *et al.* analyzed enzymatic glycosylation on a 2-cm wide biochip comprising an array of 20 peptide spots [67], and Laurent *et al.* also detected the binding of carbonic anhydrase to a benzenesulfonamide ligand and the binding of glutathione S-transferase-tagged protein complexes to a glutathione ligand [68]. Several disease-specific marker proteins in real biological samples were also identified: amyloid β-peptides [69], rat plasma profiling for biomarker discovery [70], downregulated biomarker identification [71], and liver cirrhosis protein classification [72]. At least two major limitations to the system are; (i) a bulky and sophisticated setup, and (ii) a calibration curve is necessary to quantify protein abundance. To the best of our knowledge, there is no specific report that addresses the spatial resolution for high-density biochip detection limits and mass resolution.

### 4.2. Microcantilevers

Microcantilevers or Kelvin probes were initially used for surface characterization in atomic force microscopy. They transduce specific biomolecular recognition into nanomechanical signals, which is a differential surface stress. Protein interaction causes the cantilever to deform and thus interaction can be observed by monitoring changes using a laser beam [73,74]. This method has been applied to monitor DNA-binding proteins [75], to detect the bioterrorism agent anthrax [76], to analyze biomarkers [77,78], and to weigh individual vaccinia viruses and bacteria [79].

The microcantilever array was demonstrated as a multichannel measurement method for high-throughput studies in combination with a microfluidic channel. Thompson *et al.* showed a multiplexed detection of mismatched oligonucleotides, and antigen-antibody interactions with a 100 nm spatial resolution kelvin probe device on a chip [80]. At present, several challenges remain to be resolved: (i) integration of cantilever arrays and microfluidic channel networks is still under development; and (ii) due to the delicate nature of the cantilever, performance variations are not of an acceptable standard for commercialization, currently [81].

### 4.3. Quartz Crystal Microbalance (QCM)

The QCM technique uses acoustic waves, and is a well-established technique for monitoring mass and film thickness, investigating molecular adsorption, and studying surface reactions in the monolayer range via monitoring of changes in resonant frequency. As substances are adsorbed by the QCM surface (typically a thin gold surface), the effective quartz frequency changes. Due to the piezoelectric principle, this change results in a detectable electric field.

To date, the technique has proven valuable for studying surface-related processes in liquids including protein adsorption [82,83], and various biological reactions in real-time observation [84,85]. In contrast to optical techniques, which are not sensitive to water-associated adsorbed proteins, changes in total coupled mass, including hydrodynamically coupled water provide the f-shift of the QCM. A recent extension of the technique allows the simultaneous measurement of energy dissipation and provides new insight into the protein adsorption process. Using QCM, multichannel detection is possible on a one-chip system [86].

### 4.4. Surface Plasmon Resonance (SPR) and Localized Surface Plasmon Resonance (LSPR)

The widespread use of Biacore^®^ instruments, which are based on SPR, has made the SPR one of the most reliable tools and sources for studying protein interactions, particularly in thermodynamic and kinetic analyses. The remarkable achievements of SPR analytical tools include 2-cm wide 2-dimensional micro-fluidic chip format detection [87,88]. SPR was also adapted to protein microarrays via a modification known as SPR imaging [89,90,91,92] or grating-coupled SPR [93]. SPR imaging records the reflected light for the array in a fixed single incidence angle as a function of time. It can simultaneously monitor >1000 interactions in real time (1020 spots/108 mm^2^) [94]. Very recently, Nand *et al.* monitored a cell free protein expression and measured interactions between antibody and the expressed protein using a SPRi technique. The one spot protein synthesis and protein detection was done in a high-throughput 7 × 6 spots array format [95].

In 2000, the notion of “localized” SPR, known as localized surface plasmon resonance (LSPR), was introduced [96]. Endo *et al.* has demonstrated a 76 × 26 mm chip comprising 300 spots that sensitively detect antibody-antigen interactions using the LSPR method [97]. Raphael *et al.* presented a uniform two-dimensional periodic array of gold nanoparticles with different shapes (rectangles, squares, discs and ovals) that is suitable for LPR-based biosensing platforms [98]. With current e-beam technology, it is possible to fabricate highly dense periodic individual nanoparticle arrays with sub-μm pitch for a high-throughput biochip format. The lower fabrication costs of LSPR spectroscopy, as compared to SPR-based biosensors, make it a good candidate for commercialization. However, some limitations have bottlenecked the progress of the LSPR biosensor, such as comparatively shorter linear dynamic range, and the immobilization process of nanoparticles to solid surfaces. More accurate nanomaterial fabrication with desirable design, and the preparation of LSPR-based biochips for rapid, quantitative screening are the current challenges for this technology.

### 4.5. Anomalous Reflection of Gold

**Figure 2:**
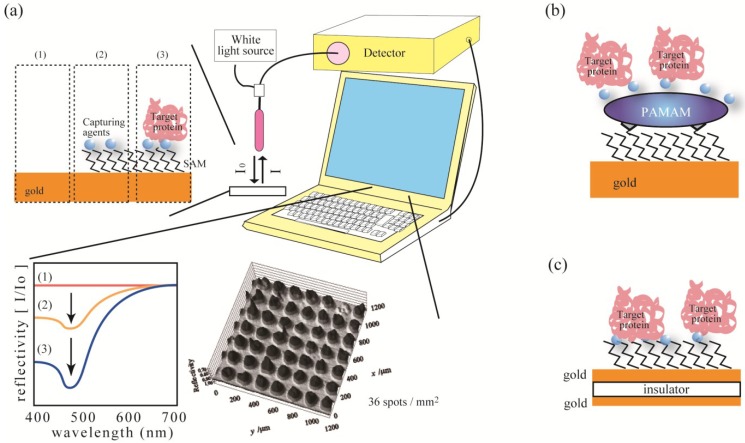
(**a**) Illustration of AR apparatus, assay images and original image data [103]. (**b**,**c**) Improvement in AR-based array technology with poly(amidoamine)dendrimer-modified gold surfaces (**b**) or gold-insulator-gold (MIM) (**c**).

Anomalous reflection (AR) of gold was introduced by Watanabe *et al.* to effectively observe biomolecular interactions (Figure 2a) [99]. When a molecular layer forms on the gold surface, significant reductions in reflectivity are observed at wavelengths of 400–500 nm. This allows the detection of molecular interactions by monitoring for changes in reflectivity. The merits of the AR technique are as follows: (i) The thickness of dielectric layer on the gold surface can be predicted using the transfer matrix technique, thus enabling quantitative measurement of surface-bound proteins [100,101,102]; (ii) unlike plasmon techniques that stringently limit the gold thin-film thickness; the use of semi-infinite gold thin-film (practically over 100 nm) is permitted for AR detection; (iii) it has tolerance in incidence angle, which allows the use of incoherent light such as light emitting diodes (LEDs); (iv) the AR technique does not require any bulky optical set up, thus making it portable and easy to miniaturize; and (v) for microarray purposes, the spatial resolution of the AR method is 10 times smaller than that of SPR. Therefore, the AR technique may provide a promising platform for high-throughput bio-molecular detection. Illumination with LED lights at normal incidence and direct collection of the reflected light made this method the most suitable for chip-format detection (49 spots/1.4 mm^2^) [103]. However, the sensitivity of the method is about one tenth that of the SPR technique. To demonstrate AR signal enhancement, Syahir *et al.* previously reported three different approaches, as follows:

In gold surface modification approach, in order to improve the sensitivity of detection of biomolecular interactions in the AR method, three-dimensional (3D) nanostructures on gold surfaces with a series of well-defined structures of poly(amidoamine) dendrimers (PAMAMs) from generation 2 to 4 (G2, G3, and G4) (Figure 2b) [104]. Surface modification using PAMAM dendrimers revealed surface roughness root mean square (RMS) values increasing from 0.708 to 4.396 for diamine (flat model surface) and the PAMAM-G4 modified surface (three-dimensional model surface), respectively. AR detection resulted in two-fold more protein (avidin) being captured on biotinylated PAMAM surfaces. For larger protein (antibiotin IgG) assay, the PAMAM G4-modified surface clearly improved the amount of proteins captured, as compared to that for the flat surface. The well-defined structure of PAMAM molecules (particularly PAMAM G4) make it possible to calculate surface expansion ratio, from flat to a three dimensional surface. We observed that the intensification of captured protein on PAMAM G4 over the diamine (flat) surface was higher than the actual surface expansion for both atomic force microscopy measurement or by theoretical means. This indicates that an appropriate surface roughness and density for capturing agents play an important role in on-surface protein interactions by lowering hindrance effects. In later experiments, it was also proposed that nm surface roughness played a more important role in molecular interactions for medium-sized proteins when compared with small or large proteins [105].

We also developed gold-containing alloy or composite approach. The use of an AR substrate with an optimized dielectric constant would improve the sensitivity when compared to that obtained with conventional gold substrates due to an optimal complex reflection coefficient at the surface [106]. Ideally, the gold substrate dielectric constant (at 470 nm wavelength) of *ε*_Au_ = −1.567 + 4.764*i* would be moved towards *ε* = −1 + *i*. Subsequently, gold-containing alloys (Au-Ag) or composites (Au-Ag_2_O) were prepared. Measurement of monomolecular layers with different thicknesses, and biotin-antibiotin IgG assay confirmed the predicted higher sensitivity as a result of dielectric constant optimization. The dielectric constants of these newly produced substrates, as measured by ellipsometer, correspond with theoretically enhanced levels of sensitivity.

In Metal-insulator-metal (MIM) approach, a new AR platform based on a metal-insulator-metal (MIM) structure (Figure 2c) has been proposed. The MIM thin-layers of Au-PMMA-Au and Au-PMMA-Ag nano-sandwich structures for the top-middle-bottom layers, respectively, were constructed [105,107]. The newly devised platform concept enables signal optimization through simple adjustment of Au or PMMA layer thicknesses empowered by computer simulations. The constructed platform had greater sensitivity and mass resolution than the previously proposed conventional AR platform. Consequently, interactions that could not be detected by conventional Au-only substrates, such as interactions between small molecules (Mw ≤ 2 kD), were successfully detected. Therein, a wide range of biological assays including small molecule detection, monosaccharide-modified peptides with surface-bound lectins, and anti-FLAG IgG with surface-bound FLAG peptides were assayed. Interactions between calmodulin protein and surface-bound peptides revealed affinity constants equivalent to those reported on affinity column analysis.

## 5. Concluding Remarks

Observing molecular events requires methods that are highly sensitive to minute changes, subsequently producing a high signal-to-noise ratio. To date, there are no general sensing platforms that can be ubiquitously applied to detect the constellation of biomolecules of various samples (saliva, urine, serum, or cell lysates) with high sensitivity, large linear dynamic range, good speed, low cost and simple design. Major limitations include problems such as: (i) time-consuming preparation steps; (ii) non-native signal interference; (iii) unsuitability for in-situ observation; (iv) necessity of sophisticated, high cost equipment; and (v) need for specialized technicians and motivated researchers to keep improving or combining principles in detection methods.

The development of label-free methods in recent years has revealed unprecedented information on molecular interactions, largely because observations are taken based on the characteristics of the biomolecules themselves. Furthermore, the issues of false positives and conjugated labels can be avoided. This could provide more reliable dynamic constant parameters that involve molecular interactions. From another perspective, detection methods that require no labeling are easily reproducible. Thus, these methods hold great potential to be further developed into a unique class of microarray analytical techniques. Nevertheless, stepping up these methods to match the commercial viability of current labeling methods will remain a challenge.

All examples given here have shown that microarray using label or label-free methods require assistance from advanced chemistry and material sciences, nano-fabrication and computational design. In the near future, it is hoped that more practical detection methods will be developed.

## References

[1] DeRisi J.L., Iyer V.R., Brown P.O. (1997). Exploring the metabolic and genetic control of gene expression on a genomic scale. Science.

[2] MacBeath G., Schreiber S.L. (2000). Printing Proteins as Microarrays for High-Throughput Function Determination. Science.

[3] Zhu H., Bilgin M., Bangham R., Hall D., Casamayor A., Bertone P., Lan N., Jansen R., Bidlingmaier S., Houfek T. (2001). Global analysis of protein activities using proteome chips. Science.

[4] Michaud G.A., Salcius M., Zhou F., Bangham R., Bonin J., Guo H., Snyder M., Predki P.F., Schweitzer B.I. (2003). Analyzing antibody specificity with whole proteome microarrays. Nat. Biotech..

[5] Batista R., Saibo N., Lourenco T., Oliveira M.M. (2008). Microarray analyses reveal that plant mutagenesis may induce more transcriptomic changes than transgene insertion. Proc. Natl. Acad. Sci. USA.

[6] Gobert G.N., McInnes R., Moertel L., Nelson C., Jones M.K., Hu W., McManus D.P. (2006). Transcriptomics tool for the human Schistosoma blood flukes using microarray gene expression profiling. Exp. Parasitol..

[7] Hsu K.L., Pilobello K.T., Mahal L.K. (2006). Analyzing the dynamic bacterial glycome with a lectin microarray approach. Nat. Chem. Biol..

[8] Tateno H., Uchiyama N., Kuno A., Togayachi A., Sato T., Narimatsu H., Hirabayashi J. (2007). A novel strategy for mammalian cell surface glycome profiling using lectin microarray. Glycobiology.

[9] Feizi T., Fazio F., Chai W., Wong C.H. (2003). Carbohydrate microarrays—A new set of technologies at the frontiers of glycomics. Curr. Opin. Struct. Biol..

[10] Phelps T.J., Palumbo A.V., Beliaev A.S. (2002). Metabolomics and microarrays for improved understanding of phenotypic characteristics controlled by both genomics and environmental constraints. Curr. Opin. Biotechnol..

[11] Park M.C., Hur J.Y., Cho H.S., Park S.H., Suh K.Y. (2011). High-throughput single-cell quantification using simple microwell-based cell docking and programmable time-course live-cell imaging. Lab Chip.

[12] Tomizaki K.-Y., Usui K., Mihara H. (2005). Protein-detecting microarrays: Current accomplishments and requirements. Chembiochem.

[13] Tomizaki K.-Y., Usui K., Mihara H. (2009). Proteins: Array-Based Techniques. Wiley Enc. Chem. Biol..

[14] Bock C., Coleman M., Collins B., Davis J., Foulds G., Gold L., Greef C., Heil J., Heilig J.S., Hicke B. (2004). Photoaptamer arrays applied to multiplexed proteomic analysis. Proteomics.

[15] Kusnezow W., Jacob A., Walijew A., Diehl F., Hoheisel J.D. (2003). Antibody microarrays: An evaluation of production parameters. Proteomics.

[16] Schweitzer B., Predki P., Snyder M. (2003). Microarrays to characterize protein interactions on a whole-proteome scale. Proteomics.

[17] Uttamchandani M., Wang J., Yao S.Q. (2006). Protein and small molecule microarrays: Powerful tools for high-throughput proteomics. Mol. Biosyst..

[18] Usui K., Takahashi M., Nokihara K., Mihara H. (2004). Peptide arrays with designed alpha-helical structures for characterization of proteins from FRET fingerprint patterns. Mol. Divers..

[19] Usui K., Tomizaki K.-Y., Mihara H. (2007). Screening of alpha-helical peptide ligands controlling a calcineurin-phosphatase activity. Bioorg. Med. Chem. Lett..

[20] Takahashi M., Nokihara K., Mihara H. (2003). Construction of a protein-detection system using a loop peptide library with a fluorescence label. Chem. Biol..

[21] Usui K., Ojima T., Tomizaki K.-Y., Mihara H. (2005). A designed glycopeptide array for characterization of sugar-binding proteins toward a glycopeptide chip technology. Nanobiotechnology.

[22] Tessier P.M., Lindquist S. (2007). Prion recognition elements govern nucleation, strain specificity and species barriers. Nature.

[23] Zhu H., Snyder M. (2001). Protein arrays and microarrays. Curr. Opin. Chem. Biol..

[24] Kodadek T. (2001). Protein microarrays: Prospects and problems. Chem. Biol..

[25] Templin M.F., Stoll D., Schwenk J.M., Potz O., Kramer S., Joos T.O. (2003). Protein microarrays: Promising tools for proteomic research. Proteomics.

[26] Tomizaki K.Y., Usui K., Mihara H. (2010). Protein-protein interactions and selection: Array-based techniques for screening disease-associated biomarkers in predictive/early diagnosis. FEBS J..

[27] de Silva A.P., Gunaratne H.Q., Gunnlaugsson T., Huxley A.J., McCoy C.P., Rademacher J.T., Rice T.E. (1997). Signaling recognition events with fluorescent sensors and switches. Chem. Rev..

[28] Dwight S.J., Gaylord B.S., Hong J.W., Bazan G.C. (2004). Perturbation of fluorescence by nonspecific interactions between anionic poly(phenylenevinylene)s and proteins: Implications for biosensors. J. Am. Chem. Soc..

[29] Usui K., Tomizaki K.Y., Mihara H. (2006). Protein-fingerprint data mining of a designed α-helical peptide array. Mol. BioSyst..

[30] Giepmans B.N., Adams S.R., Ellisman M.H., Tsien R.Y. (2006). The fluorescent toolbox for assessing protein location and function. Science.

[31] Petryayeva E., Algar W.R., Medintz I.L. (2013). Quantum dots in bioanalysis: A review of applications across various platforms for fluorescence spectroscopy and imaging. Appl. Spectrosc..

[32] Eigen M., Rigler R. (1994). Sorting single molecules: Application to diagnostics and evolutionary biotechnology. Proc. Natl. Acad. Sci. USA.

[33] Schwille P., Haupts U., Maiti S., Webb W.W. (1999). Molecular dynamics in living cells observed by fluorescence correlation spectroscopy with one- and two-photon excitation. Biophys. J..

[34] Doi N., Takashima H., Kinjo M., Sakata K., Kawahashi Y., Oishi Y., Oyama R., Miyamoto-Sato E., Sawasaki T., Endo Y. (2002). Novel fluorescence labeling and high-throughput assay technologies for *in vitro* analysis of protein interactions. Genome Res..

[35] Tomizaki K.-Y., Obi M., Mihara H. (2012). Noncompetitive on-chip immunoassays for detection of nonlabeled antibodies based on the excluded volume effect of the target itself. Bull. Chem. Soc. Jpn..

[36] Tomizaki K.-Y., Jie X., Mihara H. (2005). A chromism-based assay (CHROBA) technique for *in situ* detection of protein kinase activity. Bioorg. Med. Chem. Lett..

[37] Tomizaki K.-Y., Mihara H. (2006). Rational design of homogenous protein kinase assay platforms that allow both fluorometric and colorimetric signal readouts. Mol. Biosyst..

[38] Tomizaki K.-Y., Mihara H. (2007). Phosphate-mediated molecular memory driven by two different protein kinases as information input elements. J. Am. Chem. Soc..

[39] Yalow R.S., Berson S.A. (1959). Assay of plasma insulin in human subjects by immunological methods. Nature.

[40] Oda Y., Huang K., Cross F.R., Cowburn D., Chait B.T. (1999). Accurate quantitation of protein expression and site-specific phosphorylation. Proc. Natl. Acad. Sci. USA.

[41] Celis J.E., Gromov P. (1999). 2D protein electrophoresis: Can it be perfected?. Curr. Opin. Biotechnol..

[42] Wardeh A.J., Kay I.P., Sabaté M., Coen V.L., Gijzel A.L., Ligthart J.M., den Boer A., Levendag P.C., van Der Giessen W.J., Serruys P.W. (1999). β-Particle−emitting radioactive stent implantation: A safety and feasibility study. Circulation.

[43] Ong S.E., Blagoev B., Kratchmarova I., Kristensen D.B., Steen H., Pandey A., Mann M. (2002). Stable isotope labeling by amino acids in cell culture, SILAC, as a simple and accurate approach to expression proteomics. Mol. Cell. Proteomics.

[44] Ong S.E., Mann M. (2006). A practical recipe for stable isotope labeling by amino acids in cell culture (SILAC). Nat. Prot..

[45] Weeks I., Svehla G. (1992). Chemiluminescence Immunoassay. Wilson and Wilson’s Comprehensive Analytical Chemistry.

[46] Szkola A., Linares E.M., Worbs S., Dorner B.G., Dietrich R., Martlbauer E., Niessner R., Seidel M. (2014). Rapid and simultaneous detection of ricin, staphylococcal enterotoxin B and saxitoxin by chemiluminescence-based microarray immunoassay. Analyst.

[47] Rongen H.A., Hoetelmans R.M., Bult A., van Bennekom W.P. (1994). Chemiluminescence and immunoassays. J. Pharm. Biomed. Anal..

[48] Dodeigne C., Thunus L., Lejeune R. (2000). Chemiluminescence as diagnostic tool. A review. Talanta.

[49] Stern E., Klemic J.F., Routenberg D.A., Wyrembak P.N., Turner-Evans D.B., Hamilton A.D., LaVan D.A., Fahmy T.M., Reed M.A. (2007). Label-free immunodetection with CMOS-compatible semiconducting nanowires. Nature.

[50] Zheng G., Patolsky F., Cui Y., Wang W.U., Lieber C.M. (2005). Multiplexed electrical detection of cancer markers with nanowire sensor arrays. Nat. Biotechnol..

[51] Goda T., Miyahara Y. (2013). Label-free and reagent-less protein biosensing using aptamer-modified extended-gate field-effect transistors. Biosens. Bioelectron..

[52] Esfandyarpour R., Javanmard M., Koochak Z., Esfandyarpour H., Harris J.S., Davis R.W. (2013). Label-free electronic probing of nucleic acids and proteins at the nanoscale using the nanoneedle biosensor. Biomicrofluidics.

[53] Das J., Cederquist K.B., Zaragoza A.A., Lee P.E., Sargent E.H., Kelley S.O. (2012). An ultrasensitive universal detector based on neutralizer displacement. Nat. Chem..

[54] Romanov V., Davidoff S.N., Miles A.R., Grainger D.W., Gale B.K., Brooks B.D. (2014). A critical comparison of protein microarray fabrication technologies. Analyst.

[55] Saha K., Agasti S.S., Kim C., Li X., Rotello V.M. (2012). Gold nanoparticles in chemical and biological sensing. Chem. Rev..

[56] Hainfeld J.F., Furuya F.R. (1992). A 1.4-nm gold cluster covalently attached to antibodies improves immunolabeling. J. Histochem. Cytochem..

[57] Love J.C., Estroff L.A., Kriebel J.K., Nuzzo R.G., Whitesides G.M. (2005). Self-assembled monolayers of thiolates on metals as a form of nanotechnology. Chem. Rev..

[58] Hutter E., Fendler J.H., Roy D. (2001). Surface plasmon resonance studies of gold and silver nanoparticles linked to gold and silver substrates by 2-aminoethanethiol and 1,6-hexanedithiol. J. Phys. Chem. B.

[59] Li T., Liu D.J., Wang Z.X. (2009). Microarray-based Raman spectroscopic assay for kinase inhibition by gold nanoparticle probes. Biosens. Bioelectron..

[60] Cao Y.C., Jin R., Nam J.M., Thaxton C.S., Mirkin C.A. (2003). Raman dye-labeled nanoparticle probes for proteins. J. Am. Chem. Soc..

[61] Li T., Su M., Ma L., Liu D., Wang Z. (2014). Studying chemical-regulation of intracellular kinase activity by peptide microarray-based assay with gold nanoparticle probes. Anal. Methods.

[62] Bischoff R., Luider T.M. (2004). Methodological advances in the discovery of protein and peptide disease markers. J. Chromatogr. B.

[63] Geng X., Wang F., Li Y.G., Zhu G.P., Zhang W.M. (2007). SELDI-TOF MS proteinchip technology for screening of serum markers of HBV-induced hepatocellular carcinoma. J. Exp. Clin. Cancer Res..

[64] Kozak K.R., Amneus M.W., Pusey S.M., Su F. (2003). Identification of biomarkers for ovarian cancer using strong anion-exchange ProteinChips: Potential use in diagnosis and prognosis. Proc. Natl. Acad. Sci. USA.

[65] Malik G., Ward M.D., Gupta S.K., Trosset M.W. (2005). Serum levels of an isoform of apolipoprotein A-II as a potential marker for prostate cancer. Clin. Cancer Res..

[66] Heintz D., Wurtz V., High A.A., Van Dorsselaer A., Reski R., Sarnighausen E. (2004). An efficient protocol for the identification of protein phosphorylation in a seedless plant, sensitive enough to detect members of signalling cascades. Electrophoresis.

[67] Yeo W.-S., Min D.-H., Hsieh R.W., Greene G.L., Mrksich M. (2005). Label-free detection of protein-protein interactions on biochips. Angew. Chem. Int. Ed..

[68] Laurent N., Voglmeir J., Wright A., Blackburn J., Pham N.T., Wong S.C.C., Gaskell S.J., Flitsch S.L. (2008). Enzymatic glycosylation of peptide arrays on gold surfaces. ChemBioChem.

[69] Davies H., Lomas L., Austen B. (1999). Profiling of amyloid beta peptide variants using SELDI Protein Chip arrays. Biotechniques.

[70] Linke T., Ross A.C., Harrison E.H. (2004). Profiling of rat plasma by surface-enhanced laser desorption/ionization time-of-flight mass spectrometry, a novel tool for biomarker discovery in nutrition research. J. Chromatogr. A.

[71] Nomura F., Tomonaga T., Sogawa K., Ohashi T., Nezu M., Sunaga M., Kondo N., Iyo M., Shimada H., Ochiai T. (2004). Identification of novel and downregulated biomarkers for alcoholism by surface enhanced laser desorption/ionization-mass spectrometry. Proteomics.

[72] Xu X.Q., Leow C.K., Lu X., Zhang X., Liu J.S., Wong W.H., Asperger A., Deininger S., Eastwood Leung H.C. (2004). Molecular classification of liver cirrhosis in a rat model by proteomics and bioinformatics. Proteomics.

[73] Giljohann D.A., Mirkin C.A. (2008). Tiny tiles, tiny targets. Nat. Biotechnol..

[74] Venema L. (2007). Applied physics: Weight inside. Nature.

[75] Huber F., Hegner M., Gerber C., Guntherodt H.J., Lang H.P. (2006). Label free analysis of transcription factors using microcantilever arrays. Biosens. Bioelectron..

[76] Dhayal B., Henne W.A., Doomeweerd D.D., Reifenberger R.G., Low P.S. (2006). Detection of Bacillus subtilis spores using peptide-functionalized cantilever arrays. J. Am. Chem. Soc..

[77] Wu G., Datar R.H., Hansen K.M., Thundat T., Cote R.J., Majumdar A. (2001). Bioassay of prostate-specific antigen (PSA) using microcantilevers. Nat. Biotechnol..

[78] Arntz Y., Seelig J.D., Lang H.P., Zhang J., Hunziker P., Ramseyer J.P., Meyer E., Hegner M., Gerber C. (2003). Label-free protein assay based on a nanomechanical cantilever array. Nanotechnology.

[79] Gupta A., Akin D., Bashir R. (2004). Single virus particle mass detection using microresonators with nanoscale thickness. Appl. Phys. Lett..

[80] Thompson M., Cheran L.-E., Zhang M., Chacko M., Huo H., Sadeghi S. (2005). Label-free detection of nucleic acid and protein microarrays by scanning Kelvin nanoprobe. Biosens. Bioelectron..

[81] Boisen A., Thundat T. (2009). Design and fabrication of cantilever array sensors. Mater. Today.

[82] Höök F., Rodahl M., Kasemo B., Brzezinski P. (1998). Structural changes in hemoglobin during adsorption to solid surfaces: Effects of pH, ionic strength, and ligand binding. Proc. Natl. Acad. Sci. USA.

[83] Höök F., Rodahl M., Brzezinski P., Kasemo B. (1998). Energy dissipation kinetics for protein and antibody-antigen adsorption under shear oscillation on a quartz crystal microbalance. Langmuir.

[84] Mori T., Toyoda M., Ohtsuka T., Okahata Y. (2009). Kinetic analyses for bindings of concanavalin A to dispersed and condensed mannose surfaces on a quartz crystal microbalance. Anal. Biochem..

[85] Cooper M.A., Singleton V.T. (2007). A survey of the 2001 to 2005 quartz crystal microbalance biosensor literature: Applications of acoustic physics to the analysis of biomolecular interactions. J. Mol. Recognit..

[86] Ogi H., Nagai H., Fukunishi Y., Yanagida T., Hirao M., Nishiyama M. (2010). Multichannel wireless-electrodeless quartz-crystal microbalance immunosensor. Anal. Chem..

[87] Luo Y., Yu F., Zare R.N. (2008). Microfluidic device for immunoassays based on surface plasmon resonance imaging. Lab Chip.

[88] Safsten P., Klakamp S.L., Drake A.W., Karlsson R., Myszka D.G. (2006). Screening antibody-antigen interactions in parallel using Biacore A100. Anal. Biochem..

[89] Yeatman E., Ash E.A. (1987). Surface plasmon microscopy. Electron. Lett..

[90] Yeatman E., Ash E.A. (1988). Surface Plasmon Scanning Microscopy. SPIE Scanning Microsc. Technol. Appl..

[91] Rothenhausler B., Knoll W. (1988). Surface-plasmon microscopy. Nature.

[92] Hickel W., Kamp D., Knoll W. (1989). Surface-plasmon microscopy. Nature.

[93] Kano H., Kawata S. (1995). Grating-coupled surface plasmon for measuring the refractive index of a liquid sample. Jpn. J. Appl. Phys..

[94] Campbell C.T., Kim G. (2007). SPR microscopy and its applications to high-throughput analyses of biomolecular binding events and their kinetics. Biomaterials.

[95] Nand A., Singh V., Perez J.B., Tyagi D., Cheng Z., Zhu J. (2014). *In situ* protein microarrays capable of real-time kinetics analysis based on surface plasmon resonance imaging. Anal. Biochem..

[96] Jensen T.R., Malinsky M.D., Haynes C.L., van Duyne R.P. (2000). Nanosphere lithography: Tunable localized surface plasmon resonance spectra of silver nanoparticles. J. Phys. Chem. B.

[97] Endo T., Kerman K., Nagatani N., Hiepa H.M., Kim D.K., Yonezawa Y., Nakano K., Tamiya E. (2006). Multiple label-free detection of antigen-antibody reaction using localized surface plasmon resonance-based core-shell structured nanoparticle layer nanochip. Anal. Chem..

[98] Raphael M.P., Christodoulides J.A., Mulvaney S.P., Miller M.M., Long J.P., Byers J.M. (2012). A new methodology for quantitative LSPR biosensing and imaging. Anal. Chem..

[99] Watanabe M., Kajikawa K. (2003). An optical fiber biosensor based on anomalous reflection of gold. Sens. Actuators B Chem..

[100] Watanabe S., Usui K., Tomizaki K.Y., Kajikawa K., Mihara H. (2005). Anomalous reflection of gold applicable for a practical protein-detecting chip platform. Mol. Biosyst..

[101] Watanabe S., Tomizaki K.Y., Takahashi T., Usui K., Kajikawa K., Mihara H. (2007). Interactions between peptides containing nucleobase amino acids and T7 phages displaying *S. cerevisiae* proteins. Biopolymers.

[102] Manaka Y., Kudo Y., Yoshimine H., Kawasaki T., Kajikawa K., Okahata Y. (2007). Simultaneous anomalous reflection and quartz-crystal microbalance measurements of protein bindings on a gold surface. Chem. Commun..

[103] Fukuba S., Naraoka R., Tsuboi K., Kajikawa K. (2009). A new imaging method for gold-surface adsorbates based on anomalous reflection. Opt. Commun..

[104] Syahir A., Tomizaki K.-Y., Kajikawa K., Mihara H. (2009). Poly(amidoamine)-dendrimer-modified gold surfaces for anomalous reflection of gold to detect biomolecular interactions. Langmuir.

[105] Syahir A., Mihara H., Kajikawa K. (2010). A new optical label-free biosensing platform based on a metal-insulator-metal structure. Langmuir.

[106] Syahir A., Kajikawa K., Mihara H. (2014). Enhanced refractive index sensitivity for anomalous reflection of gold to improve performance of bio-molecular detection. Sens. Actuat. B..

[107] Syahir A., Kajikawa K., Mihara H. (2012). Sensitive detection of small molecule-protein interactions on a metal-insulator-metal label-free biosensing platform. Chem. Asian J..

